# Polycythemia Vera With Atrial Fibrillation: A Case Report and Review of the Literature

**DOI:** 10.7759/cureus.28550

**Published:** 2022-08-29

**Authors:** Waseem Umer, Naseem Umer, Elrazi A Ali, Muhammad Abubakar, Mohamed A Yassin

**Affiliations:** 1 Internal Medicine, Hamad General Hospital, Doha, QAT; 2 Medicine, Hamad Medical Corporation, Doha, QAT; 3 Internal Medicine, Hamad Medical Corporation, Doha, QAT; 4 Hematology and Oncology, Hamad General Hospital, Doha, QAT

**Keywords:** philadelphia chromosome negative, arrhythmia, mpns, atrial fibrillation, polycythemia vera

## Abstract

Polycythemia vera (PV) is a myeloid stem cell disorder characterized by excess red cell mass. The two major complications of PV are thrombosis and bleeding. Atrial fibrillation is a potentially devastating condition that can complicate the course of PV. We describe a case of a 65-year-old woman with PV who presented with new-onset atrial fibrillation. The atrial fibrillation was aborted successfully, and the patient was started on oral anticoagulation with rivaroxaban. PV with atrial fibrillation poses a challenge in management due to the risk of thrombosis and bleeding. Despite the lack of standard recommendations or guidelines, this case highlights that oral anticoagulation therapy can be safe and convenient for such patients.

## Introduction

Myeloproliferative neoplasms (MPNs) are a group of hematological malignancies characterized by an excess proliferation of myeloid cell lineages. Classically, MPN is classified as Philadelphia positive (chronic myeloid leukemia ) [1] and Philadelphia negative, which includes [2] polycythemia vera (PV), essential thrombocythemia, primary myelofibrosis, and prefibrotic myelofibrosis. Among all MPNs, PV carries the highest risk of thrombosis and cardiovascular disease. Approximately 30% to 50% of PV patients have minor or major thrombotic complications, and vascular mortality accounts for 35% to 45% of all PV-related deaths [3]. The incidence of both arterial and venous thrombosis is higher in patients with PV. The proposed mechanisms of thrombosis are multifactorial, but they involve increased shear stress due to high hematocrit leading to vessel wall inflammation and closer contact of platelets to the endothelium leading to platelet activation. Coronary events are also not uncommon in these patients; a 10-year study of 149 patients with PV found that 11.4% had a myocardial infarction (MI) [4]. Another report by Malak et al. concluded that 4% of all patients with myeloproliferative disorder die from MI [5]. Cardiac challenges in patients with PV include MI, in-stent restenosis [6], and progressive ischemic heart failure [7]. Atrial fibrillation (A-fib) is the most prevalent sustained arrhythmia, affecting up to 1% of the world's population [8]. The main complication of A-fib is thromboembolism; therefore, patients with A-fib are treated with anticoagulation. We report a PV case in a newly diagnosed A-fib patient that illustrates the challenges and management of this condition.

## Case presentation

A 65-year-old woman with a history of dyslipidemia, hypertension, PV, and heart failure with preserved ejection fraction presented to the emergency department (ED) of Hamad General Hospital with sudden onset palpitations that started two hours prior to presentation while relaxing in her room. Her symptom was associated with chest discomfort. She denied chest pain, shortness of breath, light-headedness, weakness, or abnormal sensation. She had no fever, headache, rhinorrhea, sore throat or joint pain, tremors, anxiety, weight loss, or heat intolerance. Also, she had no diarrhea, vomiting, or dizziness. A review of other systems yielded nothing remarkable. She is married, has three children, and has no previous history of miscarriage. She does not smoke, use tobacco, and does not consume alcohol, and she has never used illicit drugs.

Her PV was treated with Peginterferon alpha-2A, 135 mcg monthly, via subcutaneous injections. Her other daily medications include amlodipine/perindopril (5 mg/5 mg), aspirin 100 mg, rosuvastatin 10 mg, bisoprolol 2.5 mg, furosemide 40 mg, and amitriptyline 10 mg. There was no recent change in her regular medications.

Upon arrival at the ED, she was afebrile, and her blood pressure was 123/84 mmHg. She was tachycardic, and her heart rate ranged from 130 to 140 beats per minute (reference range: 60-100 beats per minute). She maintained 99% oxygen saturation on ambient air. She was alert and oriented with no focal neurological deficit. Her cardiovascular examination revealed irregularly irregular pulse and tachycardia with normal S1 and S2, and no added sounds were appreciated. Her jugular venous pressure was not elevated. She had no peripheral edema. The rest of the physical examination findings were unremarkable.

**Figure 1:**
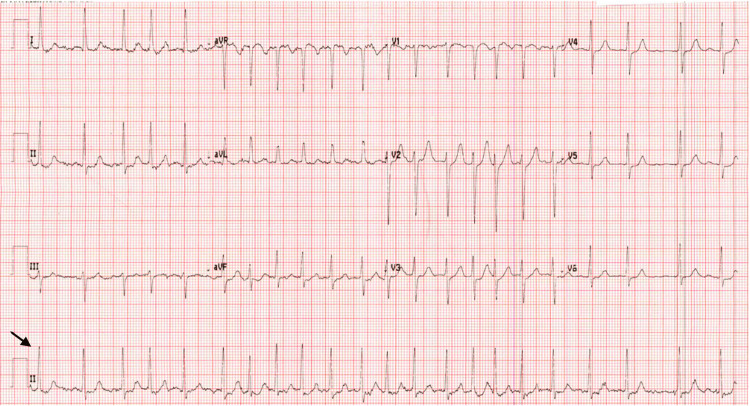
Electrocardiogram illustrating new-onset atrial fibrillation (black arrow)

Her laboratory investigations revealed elevated white blood cell count, hemoglobin, hematocrit, and reticulocyte count, and her coagulation profile and basic metabolic profile results were unremarkable (Table 1). The thyroid function test was normal. Her electrocardiography (ECG) was significant for new-onset A-fib compared to her previously recorded ECG in the records system (Figure 1). Her chest X-ray results were unremarkable. On echocardiography, she had a normal ejection fraction of 57% with grade 2 diastolic dysfunction and normal valvular function. She was treated with amiodarone infusion, which successfully restored her to normal sinus rhythm. Her CHA2DS2-VASc score (i.e., congestive heart failure, history of hypertension, age ≥75 years, diabetes, prior stroke, vascular disease, age 65 to 74, sex category) was 3 (high) and HAS BLED (i.e., hypertension, abnormal liver or renal function, stroke, bleeding, labile international normalized ratio, elderly age >65, drugs or alcohol) score was 1 (low). She was started on anticoagulation therapy with rivaroxaban 20 mg once daily, and aspirin was stopped. After a regular follow-up in our clinic for up to one year, she had no hemorrhagic or ischemic complications.

**Table 1:** Laboratory investigations on presentation to the emergency department

Analyte	Patient Values	Reference Range
Complete blood count		
White blood cell (x 10^9^/L)	20.8 x 10^9^/L	4 to 10 x 10^9^/L
Hemoglobin (g/dL)	16 g/dL	12 to 15 g/dL
Hematocrit (%)	55%	36% to 46%
Platelet count (x 10^9^/L)	221 x 10^9^/L	150 to 400 x 10^9^/L
Reticulocyte count (x 10^9^/L)	167 x 10^9^/L	50 to 100 x 10^9^/L
Coagulation profile		
Prothrombin time	11.1 seconds	9.7 to 11.8 seconds
Activated partial thromboplastin time	29.3 seconds	24.6 to 31.2 seconds
International normalized ratio	1	
Basic metabolic panel		
Urea	4.7 mmol/L	2.1 to 8.1 mmol/L
Creatinine	45 µmol/L	44 to 80 µmol/L
Sodium	137 mEq/L	135 to 145 mEq/L
Potassium	4.6 mEq/L	3.6-5.1 mEq/L
Thyroid function test		
Thyrotropin	4.04 mIU/L	0.30 to 4.2 mIU/L
Free thyroid hormone	13.9 pmol/L	11 to 23 pmol/L

## Discussion

PV is a chronic MPN characterized by an expansion of red cell mass with an increased risk of hemorrhagic and thrombotic complications [9]. The incidence of PV is approximately 1.9 per 100,000 individuals per year in the United States. The incidence rate of PV is slightly higher in men than in women (2.8 versus 1.3 cases per 100,000 per year) [10]. The condition's pathophysiology lies in the mutation in the *JAK2* (Janus kinases 2) gene in the hematopoietic stem cells (i.e., mother cells) in the bone marrow. A valine to phenylalanine substitution at position 617 of the *JAK2* gene leads to constitutively active cytokine receptors. This process leads to unregulated production of red blood cells and platelets, which results in hyperviscosity and bleeding complications. Most patients with PV are discovered incidentally when a complete blood count acquired for other reasons reveals increased hemoglobin or hematocrit. Others may present with disease-related symptoms such as headache, dizziness, visual disturbances, pruritus, early satiety, or complications such as thrombosis and bleeding. PV should be suspected in all men with hemoglobin of 16.5 g/dL or a hematocrit level of ≥49% and in women with a hemoglobin level of 16 g/dL or hematocrit level of ≥48% with normal oxygen saturation (>92% on room air) [11]. For diagnosing PV, a repeat of the complete blood count for confirmation of the results is required. Once erythrocytosis is confirmed, erythropoietin level can rule out secondary polycythemia, and, finally, *JAK2* mutation testing can confirm the PV diagnosis [11]. Finding low serum erythropoietin level and *JAK2* positivity in a patient with an elevated hemoglobin level is diagnostic of PV. Low-dose aspirin is the standard therapy for MPN patients with or without cytoreductive therapy.

According to a recent study of 37,922 patients with PV identified by the National Inpatient Sample Database, the prevalence of A-fib was 18% in PV patients compared to 11% (p<0.001) in the general population [12]. These results show some association between the two conditions, an area yet to be studied. There are no clear data on whether patients with A-fib with PV have a similar or higher risk of thrombotic-embolic complications than those with A-fib alone. Data regarding outcomes in patients with PV receiving oral anticoagulants are also limited. There is no consensus or guidelines on the best therapy for such a specialized population (e.g., low-dose aspirin, anticoagulation, or both). The therapeutic decision should be based on considering the risks and benefits along with expert opinion and experience-this is a challenge, given the high risk of bleeding and thrombosis in patients with PV with A-fib.

A review of the literature only yielded limited studies. A study in Spain compared 62 patients with PV and A-fib against 124 patients with A-fib alone. The study had a median follow-up of 2.9 and 3.7 years, respectively, and patients were treated with anticoagulation. PV patients with A-fib did not show a higher risk of thrombosis than controls [13]. A retrospective study of 437 MPN patients was conducted in Germany, from which eight patients were treated with rivaroxaban (1.8%). The data analysis revealed an odds ratio of major bleeding for patients on rivaroxaban of 1.61 (nonsignificant), which was much lower than those for patients on warfarin (1.97) [14]. A case-control study of 50 patients with MPN (essential thrombocythemia or PV) divided equally into two arms with a median follow-up of two years found a substantial difference in thrombotic or hemorrhagic events in patients treated with low-dose aspirin (control) compared with direct oral anticoagulants (DOACs; i.e., the intervention group). These results suggest that DOACs may be highly efficient and safe for use in MPN patients [15]. Based on our literature review, we started our patient on DOACs because of the reported lower risk of bleeding than warfarin in MPN patients (in addition to the liberty from routine anticoagulation monitoring with warfarin). The patient was commenced on rivaroxaban 20 mg once daily. We stopped low-dose aspirin and continued cytoreductive therapy with peginterferon. Anticoagulation therapy is challenging in patients with PV, especially with A-fib, with a paucity of literature and without the United States Food and Drug Association-approved guidelines or recommendations. Therefore, we strive to make a mutual decision that considers the risks and benefits of the therapy.

## Conclusions

Patients with PV are a high-risk population for complications from anticoagulation therapy. Though our patient managed very well, more studies on a larger scale are warranted to determine the safest options of anticoagulation in the MPN patient population with A-fib. This case highlights that need and provides evidence that oral anticoagulants could be safe in such patients.
